# Recent advances in the biosynthetic mechanisms, regulation, and detoxification strategies of deoxynivalenol in *Fusarium graminearum*

**DOI:** 10.3389/fnut.2025.1677196

**Published:** 2025-10-13

**Authors:** Mingfang He, Yaping Lei, Haiyuan Yan, Chao Sun, Xiaolong Gu

**Affiliations:** 1 Modern College of Agriculture and Forestry Engineering, Ganzhou Polytechnic, Ganzhou, China; 2 College of Food Science and Engineering, Jiangxi Agricultural University, Nanchang, China; 3 College of Veterinary Medicine, Yunnan Agricultural University, Kunming, China

**Keywords:** Fusarium head blight, deoxynivalenol (DON), TRI gene cluster, biosynthesis, regulation, detoxification

## Abstract

*Fusarium graminearum,* the major causal agent of Fusarium head blight (FHB), produces the trichothecene mycotoxin deoxynivalenol (DON), which threatens food and feed safety worldwide. This review synthesizes recent advances in DON biosynthesis, emphasizing the *TRI* gene cluster and its pathway enzymes, transcriptional regulators, and signaling cascades. In parallel, it provides a comprehensive analysis of the molecular mechanisms involved in regulating DON biosynthesis, with a focus on the *TRI* cluster. In additionally, current progress in detoxification strategies is summarized, covering physical, chemical, and biological methods aimed at mitigating DON contamination in food and feed. This review further explores the endogenous environmental factors influencing DON synthesis and offering insights to the development of integrated control strategies against DON contamination. By integrating the current findings, this review aims to support the development of effective strategies, control *F. graminearum* and mitigate FHB.

## 1 Introduction

**Figure 1:**
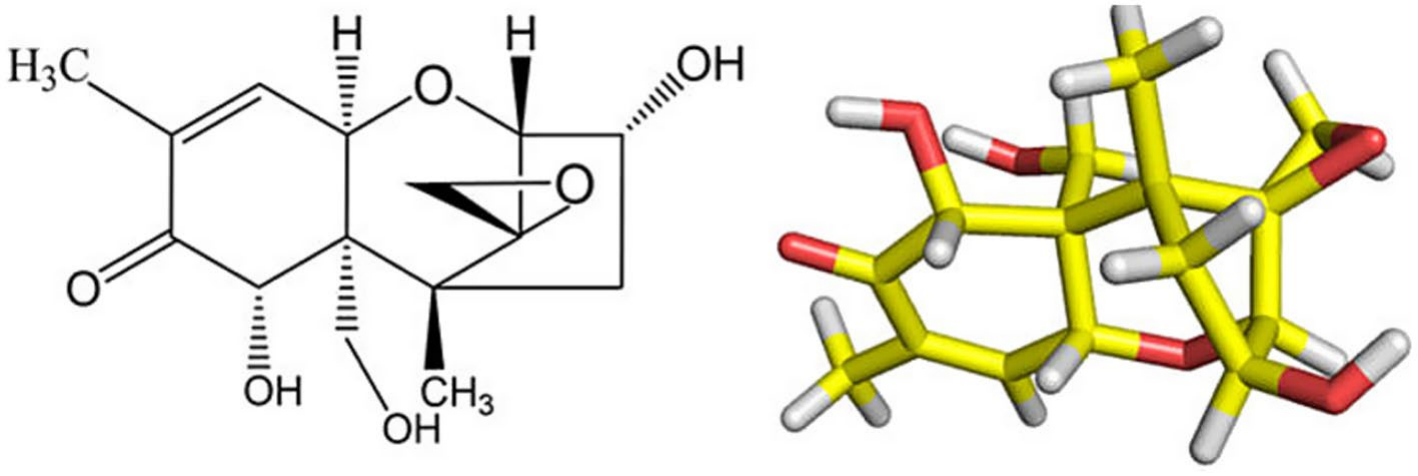
The chemical structure and stereochemical structure of DON. Key functional groups (epoxide, C9–C10 double bond) that underlie toxicity and guide detoxification chemistry are indicated.

*Fusarium graminearum* is the primary fungal pathogen responsible for FHBin wheat, a disease that threatens global grain yields and food safety (1, 2). A major concern associated with *F. graminearum* is the production of deoxynivalenol (DON) (Figure 1), or “vomitoxin,” a mycotoxin that contaminates wheat and other cereals, which poses risks to the human and animal health (3, 4). Based on the recent reports, DON is one of the most common food-related mycotoxins in the world (5). Acetylated derivatives of deoxynivalenol (DON), mainly 3-ADON and 15-ADON, act as DON precursors with slightly reduced toxicity, regulated by *FgTRI8* (6). Pathogen subspeciation has diversified their production. Deoxynivalenol-3-glucoside (D3G), a masked DON derivative, can hydrolyze *in vivo* to DON, intensifying toxicity (7). According to the Food and Agriculture Organization (FAO), approximately 25% of global food crops are contaminated with mycotoxins annually, which causes economic losses of over USD 100 billion (8, 9).

China, as one of the world’s largest wheat producers, is particularly vulnerable to FHB (10). The disease affects millions of hectares of wheat annually, with the outbreak potentially resulting in unprecedented yield losses of the nation’s wheat production (11–13). This review integrates these advances to (i) map DON biosynthesis and its regulation from gene to environment, and (ii) compare detoxification strategies with an emphasis on mechanisms, limitations, and translational prospects for integrated FHB management.

### 1.1 Geographic distribution and impact

*F. graminearum* is widely distributed across humid and semi-humid regions, with particularly high incidence in the temperate zones, including the Yangtze River Basin in China, the Great Lakes region of North America, and Central Europe (14–16). It infects over 30 cereal crops, including wheat, barley, maize, and oats (17–19).

**Figure 2:**
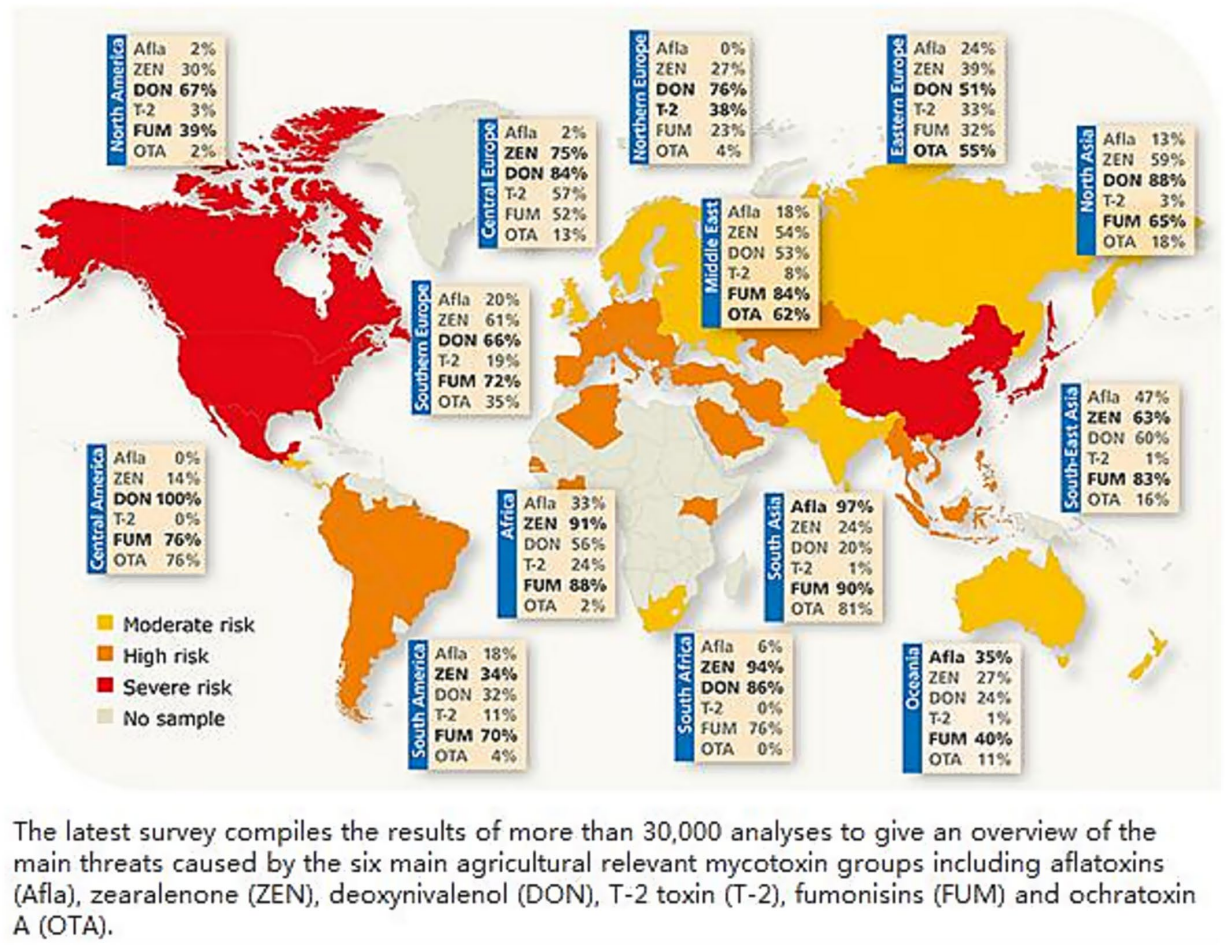
Distribution of mycotoxin contamination in the world.

According to the Biomin Global Mycotoxin Survey (Figure 2), East Asia, North America, and Europe experience the highest levels of DON contamination (20). Across the world, FHB had caused an estimated loss of over $10 billion (21). Similarly, the reported incidence of DON contamination was very high across different regions, with 94% in America and China and 77% in Europe (22, 23). DON and its acetylated derivatives exert detrimental effects on the human health, which could lead to a severe FHB outbreak and reduced wheat yields, potentially leading to multiple cases of livestock poisoningfrom DON exposure (24). Contamination levels of 3-ADON and 15-ADON are strongly correlated, and their co-occurrence produces synergistic toxicity. Studies show that baking degrades DON acetylated derivatives (25), while Juan-García et al. (26) demonstrated their cytotoxicity and metabolic products in HepG2 cells. Ozone treatment can also degrade DON, with degradation byproducts exhibiting negligible toxicity (Sun et al.) (27).

In China, FHB is most prevalent in the humid and rainy region of Yangtze River Basin, with its distribution expanding northward (28). The annual yield losses in the middle and lower Yangtze River region typically range from 10 to 15%, with severe outbreaks reducing the yields by up to 50% (29, 30). One of the earliest large-scale outbreaks occurred in Henan Province in 1985, which affected 3 million hectares of wheat fields (31). Since 2000, climate change and shifts in the cultivation practices have intensified the spread of FHB (32). Approximately 80% of wheat crops across China were contaminated to varying degrees, causing a decline in the national grain production decline of >20% (31, 33, 34). This wheat-maize rotation system has contributed to the persistence of *F. graminearum*, with Henan and Shandong Provinces emerging as high-risk areas (35).

### 1.2 Disease cycle

**Figure 3:**
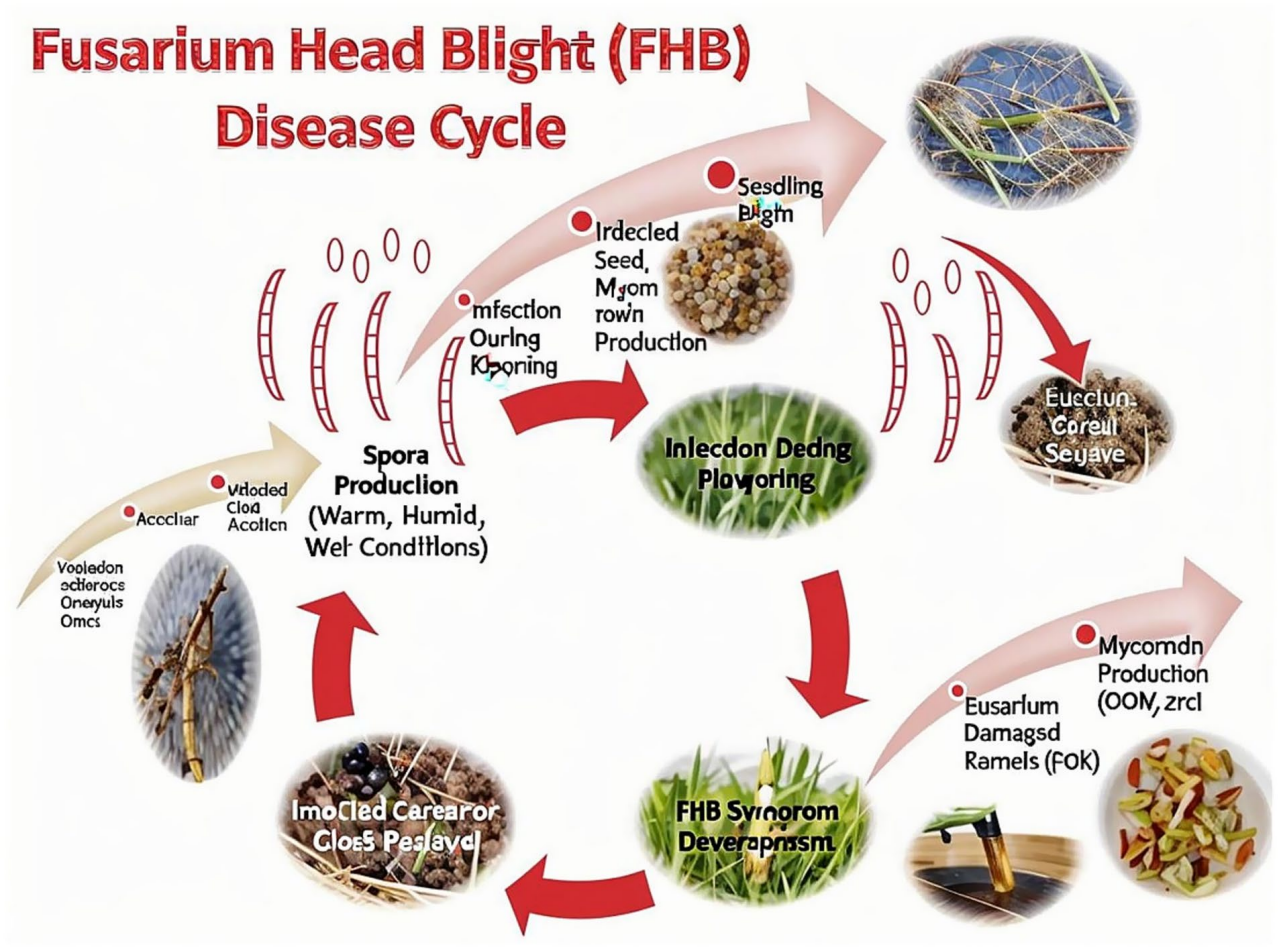
The cycle of *Fusarium graminearum* infection. Overwintering on residues, ascospore/conidial release, floret infection, and secondary spread are summarized with environmental triggers (temperature, humidity; cite from https://www.saskatchewan.ca/business/).

The *F. graminearum* comprises of the following four key stages: overwintering, spore release, infection, and secondary spread (36, 37) (Figure 3).

Regions with recurrent high DON incidence (East Asia, North America, Europe) are highlighted.

#### 1.2.1 Overwintering

Following harvest, *F. graminearum* persists as a saprophyte on crop residues, including grains and stalks, producing both mycelia and perithecia that facilitate infection in subsequent seasons (19, 38, 39). Vegetative mycelia, which is responsible for nutrient absorption, can survive on the soil surface for up to 1 year or for 1–2 months when buried (19). Dormant perithecia remain viable at soil temperatures greater than −20 C (40).

#### 1.2.2 Spore release

In spring, when temperatures reach approximately 10°C and the relative humidity is >80%, perithecia mature and release large quantities of ascospores and conidia under moist conditions (41, 42). Air currents and rain splash facilitate spore dispersal to wheat spikes, which initiate infection.

#### 1.2.3 Infection

Upon reaching wheat spikes, *F. graminearum* ascospores and conidia, forming hyphae that initially infect the anthers before spreading into the glumes and spikelets (43, 44). The infected tissues develop water-soaked brown lesions, and the fungus colonizes the spike through the rachis (45). Under warmand humid conditions, the infected spikes turn pale-yellow or white, often displaying characteristic pink or brownish fungal masses (46).

#### 1.2.4 Secondary spread

Ascospores and conidia produced on infected plants can cause secondary infections in late-season wheat crops or in the adjacent summer maize fields (47). These spores may also form perithecia on crop residues, allowing *F. graminearum* to overwinter and contribute to primary infections in the following planting season (48).

Under dry conditions, *F. graminearum* may enter a latent phase, temporarily halting its spread and symptom development (49). However, increased moisture can reactivate this disease, often causing severe epidemics (50). This observation underscores the importance of overwintering inoculum and perithecia formation in sustaining *F. graminearum* populations (19). The interplay between favorable weather conditions, abundant inoculum sources, and continuous wheat cultivation drives the recurrent nature of FHB outbreaks (51).

## 2 Biosynthesis and molecular mechanism of DON

**Figure 4:**
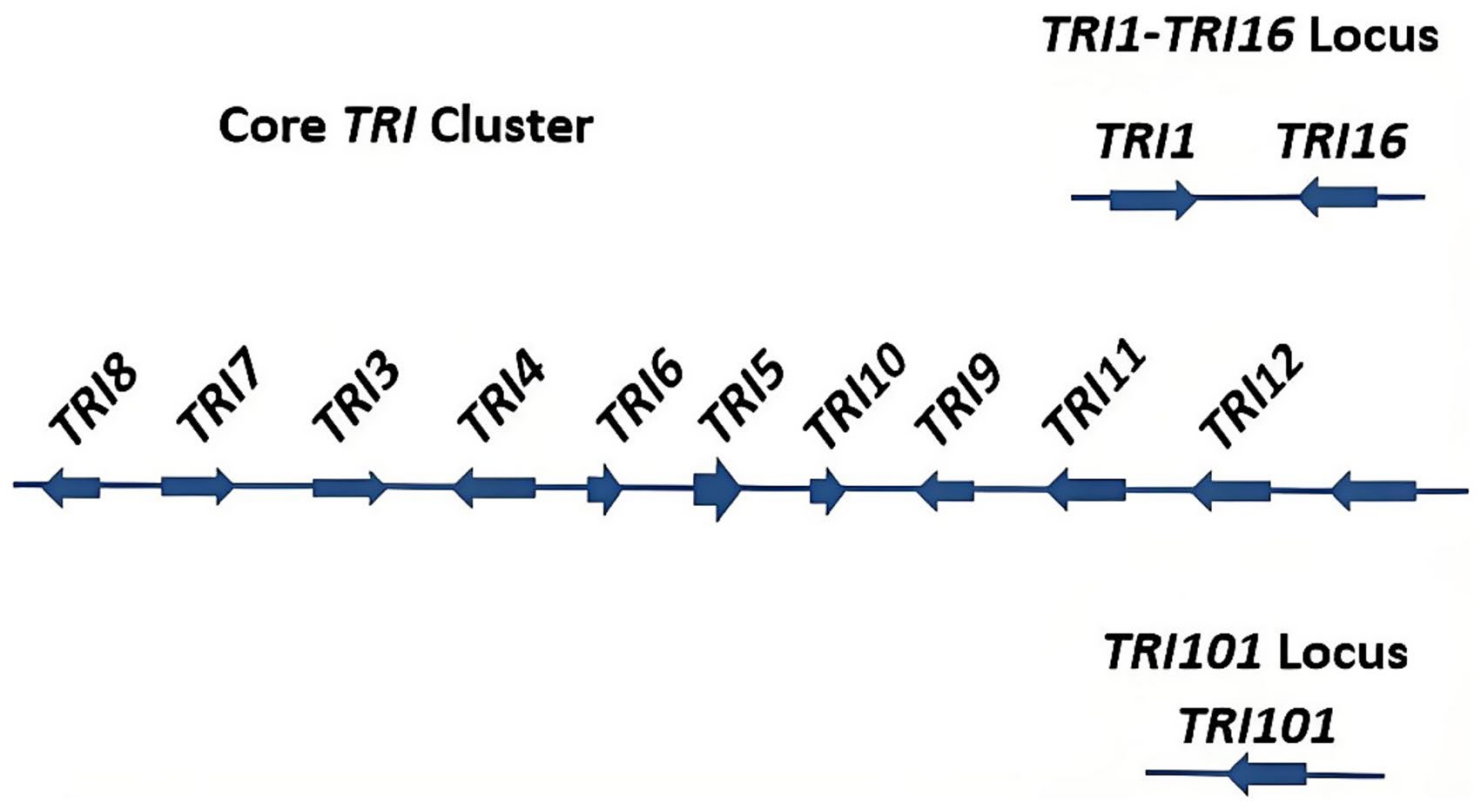
The core of TRI gene in *Fusarium graminearum*.

**Figure 5:**
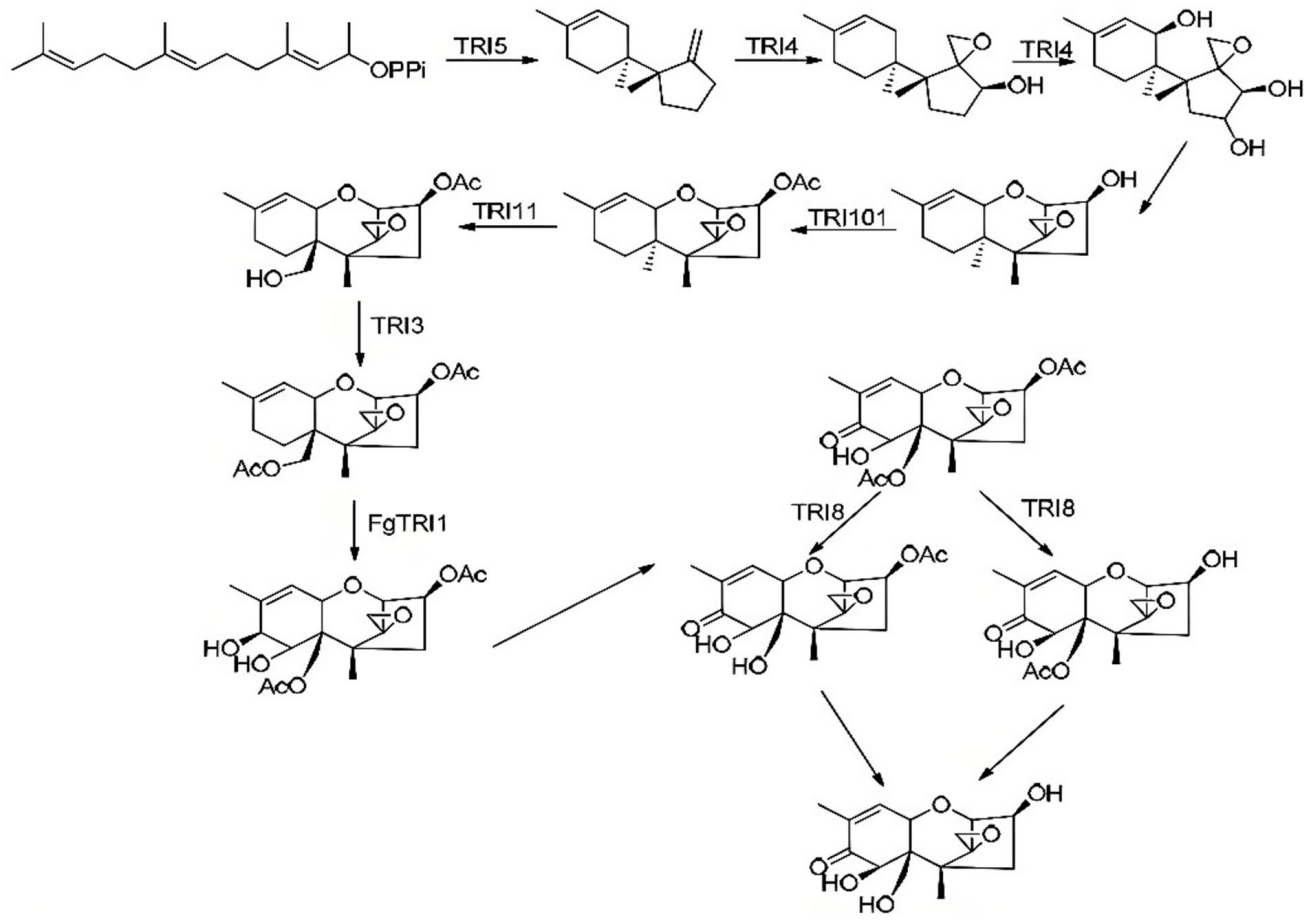
The biosynthetic pathway of DON in *Fusarium graminearum*.

The biosynthesis of DON is regulated by a coordinated enzymatic network encoded by the *TRI* family (52). All *TRI* involved in DON biosynthesis in *F. graminearum* have been identified, which includes nearly 10 core *TRI*, along with *TRI101* and *TRI1* through *TRI16* (Figure 4). The roles of these genes in DON biosynthesis have been largely elucidated, thereby clarifying the overall biosynthetic pathway (Figure 5).

Among these genes, *TRI1* encodes a C-8 hydroxylase, whereas *TRI3* encodes an acetyltransferase (53). *TRI4* encodes a cytochrome *P450* monooxygenase, and *TRI5*, also known as the trichodiene synthase gene, catalyzes the initial step in trichothecene biosynthesis (54). *TRI6* and *TRI10* function as the key regulatory genes, controlling both trichothecene biosynthesis and the expression of other *TRI* genes (55). Specifically, *TRI6* acts as a self-regulated transcription factor that modulates the *TRI10* expression through promoter binding (56). *TRI7* and *TRI13* together determine the specific chemical type of DON produced (57). Meanwhile, *TRI8* encodes a deacetylase, and *TRI12* encodes a transporter protein that serves as an efflux pump for trichothecene toxins (58, 59).

The biosynthesis process begins with the cyclization of farnesyl pyrophosphate (FPP) into the non-toxic intermediate trichodiene, catalyzed by trichodiene synthase (*TRI5*). This step is the first and most critical in DON biosynthesis. Trichodiene is subsequently hydroxylated at the C2 position, epoxidized between C12 and C13, and hydroxylated at C11 and C3 by the cytochrome P450 monooxygenase (*TRI4*), resulting in the formation of the intermediate iso-trichotriol (60, 61).

Iso-trichotriol then undergoes two non-enzymatic isomerization steps, shifting the hydroxyl group from C9 to C11 (7). A covalent bond subsequently forms between the oxygen atoms at C2 and C11, yielding iso-trichodermol, the core structure of trichothecene toxins (62). Iso-trichodermol is then acetylated at C3 by the acetyltransferase *TRI101*, forming 3-acetyl-iso-trichodermol (63). Hydroxylation at C15, catalyzed by the *TRI1*-encoded hydroxylase, produces 15-deacetylcalonectrin (54, 64). This intermediate product is further acetylated at C15 by the acetyltransferase *TRI3,* forming calonectrin (65). Additional hydroxylation at C7 and C8, followed by the conversion of the C8 hydroxyl group into a keto group, results in the formation of either 3-acetyldeoxynivalenol (3-ADON) or 15-ADON, depending on the substrate specificity of the TRI8-encoded deacetylase. The final deacetylation step yields DON (66).

As the *TRI* family directly controls DON biosynthesis, the expressions of *TRI* are central to the regulation of toxin production (67). *TRI5* was the first toxin biosynthesis gene identified and cloned in *F. graminearum*. Functional studies have demonstrated that deletion of *TRI5*, which significantly reduces the pathogenicity of *F. graminearum*, while reintroducing the wild-type gene restores both toxin production and pathogenicity (67). These gene complementation experiments not only confirmed the essential role of *TRI5* in the biosynthetic pathway but also underscored its critical position within the broader regulatory network (68).

The transcription factor *TRI6* serves as a key regulator, functioning alongside *TRI10* to control the expression of other *TRI* genes (69). Microarray analyses have demonstrated that the deletion of *TRI6* or *TRI10* affects the expression of > 50% of *TRI* genes, including *TRI12*, which encodes the toxin efflux pump (67). Beyond regulating *TRI* genes, *TRI6* and *TRI10* also influence genes involved in the mevalonate pathway, such as HMR1, which encodes 3-hydroxy-3-methylglutaryl-CoA reductase (HMG-CoA reductase), as well as genes associated with isoprenoid precursor synthesis, including those responsible for FPP production (55). The loss of *TRI6* significantly downregulates the expression of *TRI3* and *TRI4*, further disrupting DON biosynthesis (52). In addition, *TRI14* has been identified as an important regulatory gene influencing DON accumulation.

Although DON biosynthesis follows a stepwise process, it occurs within a complex metabolic network, where the sequence of individual reactions is not strictly linear (62, 70).

## 3 Regulatory mechanisms of DON biosynthesis

The regulation of DON biosynthesis is governed by both endogenous signaling pathways and external environmental factors (71, 72).

### 3.1 Regulation of DON biosynthesis by endogenous signaling pathways

Several cellular signaling pathways also contribute to the regulation of DON biosynthesis, including the mitogen-activated protein kinase (MAPK) pathway, the cyclic AMP-protein kinase A (cAMP-PKA) pathway, and the Target of Rapamycin (TOR) pathway (69).

The MAPK pathway regulates DON production through three phosphorylation cascades: Mgv1, Gpmk1, and FgHog1 (67). The deletion of core kinases in the Mgv1 pathway (i.e., FgBck1, FgMmk2, FgMgv1) severely impairs pathogenicity, restricts fungal spread to the initial infection site, and almost completely eliminates DON production (73). In the Gpmk1 pathway, the deletion of FgSte11, FgSte7, or Fgpmk1 significantly reduces both the colonization ability and DON production in wheat spikes. Similarly, the disruption of the osmotic stress-pathway genes FgHOG1, FgPBS2, and FgSSK2 significantly inhibits DON biosynthesis (13).

The cAMP-PKA pathway regulates DON biosynthesis through the catalytic subunit gene FgCPK1 and the adenylate cyclase gene FgFAC1 (74). The deletion of FgCPK1 reducesthe DON production, whereas the deletion of FgFAC1 completely abolishes DON biosynthesis (74–76).

The TOR pathway influences DON biosynthesis through FgTOR1/2, which encode the only kinases in this pathway and through the Tap42 complex genes (i.e., FgPP2A, FgSIT4, and FgPPG1). Among these, only the deletion of FgPPG1 could completely block DON biosynthesis (77, 78).

### 3.2 The influence of the external environment on DON biosynthesis

Environmental factors such as temperature, humidity, pH, carbon and nitrogen sources, hydrogen peroxide (H₂O₂), and light significantly impact the growth and metabolism of *F. graminearum*, which influence DON biosynthesis (13).

Past studies have demonstrated that *F. graminearum* can grow across a broad temperature range, with optimal DON production occurring at 22 °C –28 °C. Toxin biosynthesis can occur at 12 °C –37 °C, and the production ceases at temperatures > 37° (79). The optimal water activity (aw) for fungal growth ranges from 0.900–0.995, whereas DON biosynthesis requires a narrower range of 0.950–0.995 (80). This explains why FHB outbreaks and DON contamination are more prevalent in warm and humid regions, where the environmental conditions favor fungal proliferation and toxin production (81, 82).

Merhej et al. (83) investigated the effect of environmental pH on *F. graminearum* growth, DON biosynthesis, and *TRI* gene expression *in vitro* using liquid cultures on a minimal medium. Their results demonstrated that DON production and *TRI* expression were absent at neutral pH (84). However, by the third day of cultivation, the medium’s pH dropped sharply, triggering the expression of *TRI5* and *TRI101* and initiating accumulation of DON. Further research revealed that the transcription factor PacC, a key component of the pH regulation system, plays a critical role in secondary metabolite biosynthesis. In a follow-up study, Merhej et al. (85) reported found that the deletion of the *F. graminearum* PacC homolog (FgPAC1) led to earlier *TRI* gene induction and accelerated DON accumulation under acidic conditions, indicating that FgPAC1 could negatively regulates the *TRI* expression and DON biosynthesis (67).

Carbon and nitrogen sources, which are essential nutrients for microbial growth, also regulate DON biosynthesis in *F. graminearum*. Jiao et al. (86) analyzed the effects of 12 carbon sources on DON and 3-ADON production in nine 3-ADON-producing strains of *F. graminearum*. Sucrose, raffinose, and stachyose significantly enhanced trichothecene production across all tested strains. In sucrose-based media, the expression of *TRI4* and *TRI5* were significantly upregulated, whereas this effect was absent in glucose-based media (87). Furthermore, adding glucose to sucrose-based media did not inhibit DON accumulation, suggesting that trichothecene biosynthesis is not regulated by carbon catabolite repression (88). Instead, *F. graminearum* appears to directly recognize sucrose molecules, activating *TRI* expressions and initiating the trichothecene biosynthesis pathway (67).

In terms of nitrogen sources, guanidino-butyrate, arginine, and ornithine strongly induce DON biosynthesis, whereas ammonium, nitrate, leucine, and tyrosine exhibit inhibitory effects (89). The nitrogen metabolic regulator gene FgAREA is induced under nitrogen-limiting conditions, which promotes secondary nitrogen utilization and activates the expression of *TRI*, including *TRI5*, *TRI6*, and *TRI10* (90). In contrast, FgNMR1, a co-repressor involved in nitrogen catabolite repression, inhibits FgAREA under nitrogen-sufficient conditions, thereby suppressing DON biosynthesis (91). However, the deletion of FgNMR1 alone does not significantly affect DON production (92).

Other chemical compounds can also influence DON biosynthesis. For example, H₂O₂ has been demonstrated to enhance DON and 15-ADON production by activating the *TRI* expression, particularly TRI4, *TRI5*, and *TRI12* (93). In contrast, adding catalase to cultures could significantly reduce *TRI* expression and DON accumulation. This regulatory effect is associated with oxidative stress-responsive transcription factors, including FgAP1, FgATF1, and FgSKN7. The deletion of FgSKN7 significantly reduced DON biosynthesis and impaired H₂O₂-induced *TRI* expression (94). Interestingly, the deletion of FgAP1 increased the DON production and enhanced the *TRI* expression, suggesting that the loss of FgAP1 disrupts oxidative stress regulation and triggers abnormal *TRI* overexpression (95).

In addition, ferulic acid has been exhibited to suppress *TRI* expression and reduce DON biosynthesis through transcriptional regulation (96). Boutigny et al. (97) demonstrated that DON production is inversely correlated with the initial ferulic acid concentration in the medium, with higher concentrations exerting stronger inhibitory effects.

## 4 Advances in the detoxification and control of DON

DON is chemically stable and highly resistant to heat, acidic conditions, and long-term storage, making its elimination difficult through the conventional processing methods. Therefore, the development of efficient, safe, and cost-effective detoxification strategies deemed is critical for ensuring food and feed safety. The current methods for mitigating DON contamination fall into three main categories: physical, chemical, and biological approaches.

The physical methods aim to remove or inactivate DON through techniques such as sorting, adsorption, irradiation, or thermal processing. Chemical methods involve the use of reagents, such as alkalis, ozone, or oxidants,to alter the molecular structure of DON and reduce its toxicity. Biological methods rely on microorganisms, enzymes, or plant metabolic pathways to adsorb, degrade, or transform DON. Among these, biological strategies are particularly promising owing to their mild operational conditions, high specificity, environmental sustainability, and ability to preserve the nutritional quality.

### 4.1 Physical methods

#### 4.1.1 Thermal processing

Thermal treatment is the most commonly applied physical approach for reducing DON contamination. In general, higher temperatures yield better detoxification efficiency. The common techniques include steaming, baking, frying, canning, and extrusion (7). For instance, superheated steam treatment at 185 °C for 6 min reduced the DON levels in contaminated wheat by 52% (98, 99). Frying at 169–243 °C decreased DON concentrations in wheat dough by 20–28%, whereas baking of bread led to 54% of 82% reduction (100). Despite these promising results, the mechanisms behind DON reduction during heating remain unclear. It remains unknown whether DON is fully degraded or simply adsorbed onto the food matrix. Moreover, the identity and toxicity of the resulting degradation products are not well characterized. Advantages—well-established technology with proven scalability; Limitations—the mechanisms of action and the toxicity of degradation products remain incompletely elucidated, and potential impacts on quality and nutritional attributes cannot be excluded.

#### 4.1.2 Irradiation

Three primary irradiation techniques have been investigated in relation to DON degradation: gamma irradiation, electron beam irradiation, and ultraviolet (UV) irradiation. Khaneghah et al. (101) reported found that the efficiency of DON degradation by electron beam irradiation increased with higher doses, which was also influenced by the concentration of DON in the solution. Specifically, at doses of 1–10 kGy, higher solution concentrations resulted in greater degradation, whereas the detoxification rate of DON in the aqueous solution was 89.13% at 20 kGy (102). Irradiation exhibited greater effectiveness in aqueous environments, with little effect on DON in dry materials such as wheat and corn, limiting its applicability to solid commodities.

DON is also sensitive to UV light. Feizollahi et al. (103) demonstrated that UV irradiation significantly degraded DON, with enhanced efficacy detected under longer exposure times, shorter irradiation distances, and lower solution pHs. Shanakhat et al. (104) performed UV irradiation at 254 nm for 15, 30, 60, and 120 min on semolina to reduce the mycotoxin contamination. In fact, UV irradiation has been widely explored for degrading aflatoxins, albeit its application to DON remains limited. Moreover, the inconsistent performance, shallow penetration depth, and the potential to damage sensitive nutrients such as vitamins significantly constrain its practical utility in DON detoxification. Advantages—high degradation efficiency in aqueous systems; Limitations—poor penetration in solid matrices, narrow parameter windows, and unfavorable effects on sensitive nutrients.

#### 4.1.3 Adsorption

A range of adsorbents is currently available in the market for DON removal, including activated carbon, inorganic aluminosilicates such as hydrated sodium calcium aluminosilicate (HSCAS), and organic materials such as glucomannan and yeast cell walls. Activated carbon can adsorb and remove 90.5% of DON and AFB1 (105). A newly developed composite adsorbent of HSCAS achieved an average DON adsorption rate of 90% (106). However, these adsorption method has several limitations. It often requires elevated temperatures or stringent conditions, and may non-selectively bind essential micronutrients in the food or feed. Furthermore, if DON is only adsorbed but not degraded, there is a risk of secondary contamination. Considering such concerns, the European Union does not permit the use of adsorbents for mycotoxin mitigation in animal feed. As such, adsorption is not considered the most reliable strategy for DON detoxification. Advantages—simple implementation and low cost; Limitations—lack of selectivity, concomitant adsorption of nutrients, risk of recontamination, and restricted regulatory acceptance.

### 4.2 Chemical methods

Chemical degradation methods involve the breakdown of functional groups on the DON molecule through exposure to strong acids, bases, or oxidants, with the aim of ultimately reducing or eliminating its toxicity. Common techniques employed for this include alkaline hydrolysis, ammoniation, and oxidation. DON is particularly sensitive to alkaline conditions and readily degrades in basic solutions. Treatment of DON-contaminated wheat with sodium carbonate (Na₂CO₃) and sodium bisulfite (NaHSO₃) yielded DON-reduction rates of 83.9 and 69.9%, respectively (7). These methods are most effective for high-moisture materials, such as silage and liquid fats, but less suitable for solid feeds such as oilseed cakes or bulk feed ingredients.

**Figure 6:**
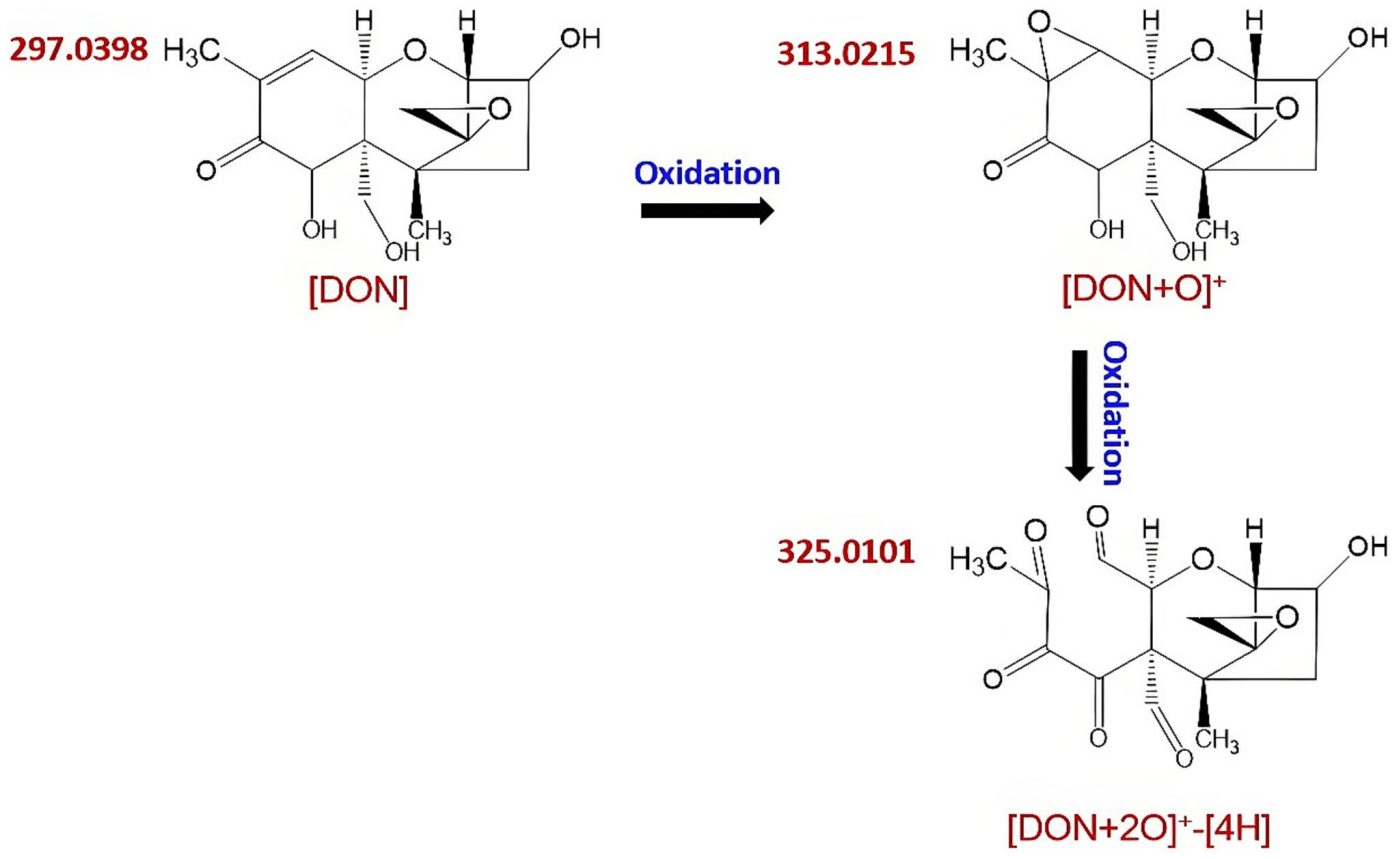
The chemical process of DON and ozone.

Ozone is a powerful oxidizing agent that can rapidly cleave double bonds in organic compounds. It exhibits excellent penetration ability and readily decomposes into oxygen without leaving any toxic residues. Moreover, ozone is easy to generate on-site, requires no storage or post-treatment, and has been widely recognized by researchers globally for its remarkable potential in practical applications. Among oxidants, ozone has received growing attention due to its strong oxidative potential. It targets the C9–C10 double bond in DON’s structure, breaking it down into simpler, less toxic compounds such as acids, aldehydes, and ketones (107, 108). As illustrated in Figure 6, ozone reacts directly with the molecular structure of DON (109). In recent years, ozone has emerged as a widely studied and applied technique for controlling fungal growth and mycotoxin contamination in diverse food products. It effectively kills harmful microbes and insects, reduces pesticide residues, and extends the shelf life of stored grains.

Young et al. (108) demonstrated that ozone treatment effectively degraded DON in wheat and corn, with significantly better outcomes observed in humid ozone environments relative to that in dry ozone. Additionally, Yang et al. (110), based on their investigation of the degradation efficiency of ozonated water at different concentrations on the trichothecene mycotoxins, proposed preliminary pathways for the formation of degradation products. Their findings consistently indicated that ozone was highly effective in degrading DON.

Obadi et al. (111) reported that ozone reacts with double bonds in carotenoid-like compounds, resulting in the reduction of the yellowness of flour and an increase in brightness. Ozone also reacts with the double bonds of unsaturated fatty acids, generating free radicals that can cause rancidity. In addition, ozone exposure was found to alter the gelatinization properties of starch. Bamyar et al. (112) further demonstrated that moderate ozone treatment enhanced the dough strength of wheat flour and reduced its extensibility; however, excessive ozone treatment led to a decrease in the ratio of unextractable polymeric protein to extractable polymeric protein (UPP/EP), indicating a potential degradation of the gluten quality.

Therefore, when applying ozone technology to degrade mycotoxins, it is essential to evaluate its impact on the nutritional value and the processing quality of grains. Presently, studies assessing the nutritional properties of major DON-contaminated commodities such as wheat and corn after ozone treatment remain limited. This lack of a comprehensive quality-evaluation system for ozone-treated raw materials directly restricts the broader application and commercialization of ozone detoxification technologies. Advantages—high efficiency with *in situ* generation, well-defined reactivity toward double bonds; Limitations—requires careful evaluation of impacts on dough rheology, lipid oxidation, color, and other quality parameters.

### 4.3 Biological methods

Despite the limited reports, recent studies both domestically and internation-ally, have demonstrated a significant progress in the biological degradation of DON. Microorganisms can secrete extracellular enzymes that catalyze various chemical reactions—such as de-epoxidation, deacetylation, hydroxylation, hydrolysis, and glycosylation—to convert DON into less toxic metabolites.

For example, Wang et al. (113) isolated a bacterial strain from soil that could use DON as its sole carbon source, which achieved a degradation efficiency of 63%. Liu et al. (114) screened and identified an effective DON-degrading *Bacillus* strain, which, when added to animal feed, reduced the DON levels by up to 50.69%.

Enzymatic degradation methods, particularly, offer high specificity and efficiency by exploiting the unique substrate affinity of enzymes to catalyze mycotoxin breakdown. These methods prevent toxin regeneration and are highly selective. Chen et al. (115) demonstrated that enzymes produced by *Gordonia hydrophobica* HAU421 could cleave the epoxide ring of trichothecenes, thereby significantly reducing the toxicity of DON.

Despite these advantages, the existing biological methods face several limitations. Their economic feasibility is low, and the inherent microbial activity fluctuates with the environmental conditions. The degradation process is typically slow, and it is challenging to apply these methods to solid matrices. Furthermore, the safety and composition of microbial metabolites are often difficult to evaluate. As a result, the presently known practical application of biological detoxification strategies for DON remains limited. Advantages—high specificity, mild conditions, and potential for selective biotransformation; Limitations—ladaptation to solid matrices and industrial-scale application remain challenging, and the safety of metabolic products requires systematic evaluation.

## 5 Conclusion

This review consolidates mechanistic insights into DON biosynthesis and its regulation, emphasizing the TRI cluster, transcriptional control, signaling cross-talk, and environmental modulation. While pathway enzymes and key regulators are increasingly well mapped, context-dependent TRI expression and matrix-specific detoxification efficacy remain major sources of variability.

Currently, chemical fungicides are widely being used to manage FHB during agricultural production. However, concerns regarding chemical residues and environmental contamination underscore the urgent need for eco-friendly control strategies. A deeper understanding of the biosynthetic and regulatory mechanisms governing DON production in *F. graminearum* provides a crucial foundation for developing more sustainable disease management approaches.

Each detoxification strategy presents unique strengths and limitations. The known physical methods are scalable but may leave toxic residues. Chemical approaches are effective but can damage product quality or pose safety risks. Biological methods offer specificity and sustainability but are constrained by process complexity and scalability. Therefore, the choice of detoxification method should consider not only efficacy but also safety, regulatory compliance, and compatibility with food/feed matrices.

## 6 Prospects

Future research should focus on leveraging gene editing technologies to enhance the endogenous resistance to DON and breeding new wheat cultivars with both FHB resistance and reduced toxin accumulation. In parallel, the hybrid methods, such as combining physical, chemical, and biological techniques, to maximize the detoxification efficiency. Innovations in enzyme engineering and microbial synthetic biology may yield more robust strains and catalytic tools. Moreover, regulatory frameworks must evolve to evaluate detoxification products comprehensively and guide the safe implementation of biological methods in the food industry.

These advancements of advancements in gene editing, enzyme engineering, and hybrid detoxification methods could offer promising solutions for mitigating FHB outbreaks and ensuring global food security.

## References

[1] He WJ Yang P Huang T Liu YF Zhang YW Zhang WM . Detoxifying bacterial genes for deoxynivalenol epimerization confer durable resistance to Fusarium head blight in wheat. Plant Biotechnol J. (2024) 22:2395–409. doi: 10.1111/pbi.14353, PMID: 38593377 PMC11331793

[2] Zhang H Zhang B He H Zhang L Hu X Wu C. Evaluation of wheat grain and processing quality under fusarium head blight control using strong oxidizing radicals. Foods. (2025) 14:1236. doi: 10.3390/foods14071236, PMID: 40238449 PMC11988342

[3] Chen L Yang J Wang H Yang X Zhang C Zhao Z . NX toxins: new threat posed by *Fusarium graminearum* species complex. Trends Food Sci Technol. (2022) 119:179–91. doi: 10.1016/j.tifs.2021.11.027

[4] Tian Y Zhang D Cai P Lin H Ying H Hu Q-N . Elimination of *Fusarium* mycotoxin deoxynivalenol (DON) via microbial and enzymatic strategies: current status and future perspectives. Trends Food Sci Technol. (2022) 124:96–107. doi: 10.1016/j.tifs.2022.04.002

[5] Zha A Tu R Cui Z Qi M Liao S Wang J . Baicalin–zinc complex alleviates inflammatory responses and hormone profiles by microbiome in deoxynivalenol induced piglets. Front Nutr. (2021) 8:738281. doi: 10.3389/fnut.2021.738281, PMID: 34692749 PMC8534294

[6] Guo H Ji J Wang J s Sun X. Deoxynivalenol: masked forms, fate during food processing, and potential biological remedies. Compr Rev Food Sci Food Saf. (2020) 19:895–926. doi: 10.1111/1541-4337.12545, PMID: 33325179

[7] Feizollahi E Roopesh M. Mechanisms of deoxynivalenol (DON) degradation during different treatments: a review. Crit Rev Food Sci Nutr. (2022) 62:5903–24. doi: 10.1080/10408398.2021.1895056, PMID: 33729830

[8] Eskola M Kos G Elliott CT Hajšlová J Mayar S Krska R. Worldwide contamination of food-crops with mycotoxins: validity of the widely cited ‘FAO estimate’of 25%. Crit Rev Food Sci Nutr. (2020) 60:2773–89. doi: 10.1080/10408398.2019.1658570, PMID: 31478403

[9] Gómez-Salazar JA Ruiz-Hernández K Martínez-Miranda MM Castro-Ríos K. Postharvest strategies for decontamination of aflatoxins in cereals. Food Rev Int. (2023) 39:3635–62. doi: 10.1080/87559129.2021.2013254

[10] Hu C Chen P Zhou X Li Y Ma K Li S . Arms race between the host and pathogen associated with Fusarium head blight of wheat. Cells. (2022) 11:2275. doi: 10.3390/cells11152275, PMID: 35892572 PMC9332245

[11] King J Dreisigacker S Reynolds M Bandyopadhyay A Braun HJ Crespo-Herrera L . Wheat genetic resources have avoided disease pandemics, improved food security, and reduced environmental footprints: a review of historical impacts and future opportunities. Glob Chang Biol. (2024) 30:e17440. doi: 10.1111/gcb.17440, PMID: 39185562

[12] Wang H Sun S Ge W Zhao L Hou B Wang K . Horizontal gene transfer of Fhb7 from fungus underlies Fusarium head blight resistance in wheat. Science. (2020) 368:eaba5435 2020. doi: 10.1126/science.aba5435, PMID: 32273397

[13] Xu M Wang Q Wang G Zhang X Liu H Jiang C. Combatting Fusarium head blight: advances in molecular interactions between *Fusarium graminearum* and wheat. Phytopathol Res. (2022) 4:37. doi: 10.1186/s42483-022-00142-0

[14] Kaul N Kashyap PL Kumar S Singh D Singh GP. Genetic diversity and population structure of head blight disease causing fungus *Fusarium graminearum* in northern wheat belt of India. J Fungi. (2022) 8:820. doi: 10.3390/jof8080820, PMID: 36012808 PMC9409692

[15] Pradhan M. P. (2011). Combining Fusarium head blight resistance and barley yellow dwarf virus tolerance in spring wheat (*Triticum aestivum* L.) University of Manitoba (Canada).

[16] Song Y Linderholm HW Wang C Tian J Huo Z Gao P . The influence of excess precipitation on winter wheat under climate change in China from 1961 to 2017. Sci Total Environ. (2019) 690:189–96. doi: 10.1016/j.scitotenv.2019.06.367, PMID: 31288110

[17] Alisaac E Mahlein A-K. Fusarium head blight on wheat: biology, modern detection and diagnosis and integrated disease management. Toxins. (2023) 15:192. doi: 10.3390/toxins15030192, PMID: 36977083 PMC10053988

[18] Goswami RS Kistler HC. Heading for disaster: *Fusarium graminearum* on cereal crops. Mol Plant Pathol. (2004) 5:515–25. doi: 10.1111/j.1364-3703.2004.00252.x, PMID: 20565626

[19] Leplat J Friberg H Abid M Steinberg C. Survival of *Fusarium graminearum*, the causal agent of Fusarium head blight. A review. Agron Sustain Dev. (2013) 33:97–111. doi: 10.1007/s13593-012-0098-5

[20] Chelós J. Tolosa Carrasco Y. Rodríguez Leal M. J. Ruiz Donat P. Vila (2021). Multi-mycotoxin occurrence in feed, metabolism and carry-over to animal-derived food products: a review. 10.1016/j.fct.2021.112661 34762978

[21] Joshi P Sandhu KS Dhillon GS Chen J Bohara K. Detection and monitoring wheat diseases using unmanned aerial vehicles (UAVs). Comput Electron Agric. (2024) 224:109158. doi: 10.1016/j.compag.2024.109158

[22] Mishra S Srivastava S Dewangan J Divakar A Rath SK. Global occurrence of deoxynivalenol in food commodities and exposure risk assessment in humans in the last decade: a survey. Crit Rev Food Sci Nutr. (2022) 60:1346–74. 10.1080/10408398.2019.1571479 30761910

[23] U. Human: Biomin world mycotoxin survey 2018. AFMA Matrix, 28, 16–19 (2019)

[24] Ganesan AR Mohan K Rajan DK Pillay AA Palanisami T Sathishkumar P . Distribution, toxicity, interactive effects, and detection of ochratoxin and deoxynivalenol in food: a review. Food Chem. (2022) 378:131978. doi: 10.1016/j.foodchem.2021.131978, PMID: 35033712

[25] Wu L Wang B. Evaluation on levels and conversion profiles of DON, 3-ADON, and 15-ADON during bread making process. Food Chem. (2015) 185:509–16. doi: 10.1016/j.foodchem.2015.03.082, PMID: 25952900

[26] Juan-García A Taroncher M Font G Ruiz M-J. Micronucleus induction and cell cycle alterations produced by deoxynivalenol and its acetylated derivatives in individual and combined exposure on HepG2 cells. Food Chem Toxicol. (2018) 118:719–25. doi: 10.1016/j.fct.2018.06.024, PMID: 29908960

[27] Sun C Mao C Zhou Z Xiao J Zhou W Du J . In vitro assessment of ozone-treated deoxynivalenol by measuring cytotoxicity and wheat quality. Toxins. (2024) 16:64. doi: 10.3390/toxins16020064, PMID: 38393142 PMC10893320

[28] Ji L Li Q Wang Y Burgess LW Sun M Cao K . Monitoring of *Fusarium* species and trichothecene genotypes associated with Fusarium head blight on wheat in Hebei Province, China. Toxins. (2019) 11:243. doi: 10.3390/toxins11050243, PMID: 31035348 PMC6563079

[29] Cui L Selvaraj JN Xing F Zhao Y Zhou L Liu Y. A minor survey of deoxynivalenol in *Fusarium* infected wheat from Yangtze–Huaihe river basin region in China. Food Control. (2013) 30:469–73. doi: 10.1016/j.foodcont.2012.08.011

[30] Xu M Xu Y Xu J Xu M Yang R Liu X. Construction of a comprehensive meteorological risk index for wheat *Fusarium* head blight prediction based on more than a half-century of monitoring data. Plant Dis. (2025) 109:698–708. doi: 10.1094/PDIS-05-24-1095-RE, PMID: 39412845

[31] Qiu J Xu J Shi J. Fusarium toxins in Chinese wheat since the 1980s. Toxins. (2019) 11:248. doi: 10.3390/toxins11050248, PMID: 31052282 PMC6562770

[32] Jung J-Y Kim J-H Baek M Cho C Cho J Kim J . Adapting to the projected epidemics of *Fusarium* head blight of wheat in Korea under climate change scenarios. Front Plant Sci. (2022) 13:1040752. doi: 10.3389/fpls.2022.1040752, PMID: 36582642 PMC9793406

[33] Chen C Frank K Wang T Wu F. Global wheat trade and codex alimentarius guidelines for deoxynivalenol: a mycotoxin common in wheat. Glob Food Secur. (2021) 29:100538. doi: 10.1016/j.gfs.2021.100538

[34] Li S Liu N Cai D Liu C Ye J Li B . A predictive model on deoxynivalenol in harvested wheat in China: revealing the impact of the environment and agronomic practicing. Food Chem. (2023) 405:134727 36335729 10.1016/j.foodchem.2022.134727

[35] Gall E Benkeblia N. Mycoagroecology: Integrating fungi into agroecosystems. Boca Raton, FL, USA: CRC Press (2022).

[36] Brewer H. C. (2015). Functional evaluation of plant defence signalling against *Fusarium graminearum* and *F. culmorum* in Arabidopsis floral tissue. University of Exeter (United Kingdom).

[37] Bergstrom GC Nicholson RL. The biology of corn anthracnose: knowledge to exploit for improved management. Plant Dis. (1999) 83:596–608. doi: 10.1094/PDIS.1999.83.7.596, PMID: 30845609

[38] Kelly L. (2018). *Fusarium* species associated with grain sorghum and mungbean in Queensland.

[39] Trail F. For blighted waves of grain: *Fusarium graminearum* in the postgenomics era. Plant Physiol. (2009) 149:103–10. doi: 10.1104/pp.108.129684, PMID: 19126701 PMC2613717

[40] Ngolong Ngea GL Yang Q Xu M Ianiri G Dhanasekaran S Zhang X . Revisiting the current and emerging concepts of postharvest fresh fruit and vegetable pathology for next-generation antifungal technologies. Compr Rev Food Sci Food Saf. (2024) 23:e13397. doi: 10.1111/1541-4337.13397, PMID: 38924311

[41] Manstretta V Morcia C Terzi V Rossi V. Germination of *Fusarium graminearum* ascospores and wheat infection are affected by dry periods and by temperature and humidity during dry periods. Phytopathology. (2016) 106:262–9. doi: 10.1094/PHYTO-05-15-0118-R, PMID: 26623994

[42] Beyer M Verreet J-A Ragab WS. Effect of relative humidity on germination of ascospores and macroconidia of *Gibberella zeae* and deoxynivalenol production. Int J Food Microbiol. (2005) 98:233–40. doi: 10.1016/j.ijfoodmicro.2004.07.005, PMID: 15698684

[43] Kumar S Kashyap PL Singh GP. Wheat blast. Boca Raton, FL, USA: CRC Press (2020).

[44] Guenther J. C. (2003). The colonization of wheat stems and subsequent perithecium development by Gibberella zeae (*Anamorph Fusarium graminearum*) Michigan State University.

[45] Kheiri A Moosawi Jorf SA Malihipour A. Infection process and wheat response to Fusarium head blight caused by *Fusarium graminearum*. Eur J Plant Pathol. (2019) 153:489–502. doi: 10.1007/s10658-018-1576-7

[46] Shay R Wiegand AA Trail F. Biofilm formation and structure in the filamentous fungus *Fusarium graminearum*, a plant pathogen. Microbiol Spect. (2022) 10:e00171–22. doi: 10.1128/spectrum.00171-22, PMID: 35950855 PMC9430603

[47] Murray TD. Diseases of small grain cereal crops: a colour handbook. Boca Raton, FL: CRC Press, Taylor & Francis Group (2013).

[48] Manstretta V Rossi V. Effects of temperature and moisture on development of *Fusarium graminearum* perithecia in maize stalk residues. Appl Environ Microbiol. (2016) 82:184–91., PMID: 26475114 10.1128/AEM.02436-15 PMC4702647

[49] Vurro M Bonciani B Vannacci G. Emerging infectious diseases of crop plants in developing countries: impact on agriculture and socio-economic consequences. Food Secur. (2010) 2:113–32. doi: 10.1007/s12571-010-0062-7

[50] Bigini V Sillo F Giulietti S Pontiggia D Giovannini L Balestrini R . Oligogalacturonide application increases resistance to *Fusarium* head blight in durum wheat. J Exp Bot. (2024) 75:3070–91. doi: 10.1093/jxb/erae050, PMID: 38334507

[51] Park J Jeon H Hwangbo A Min K Ko J Kim J-E . A winged-helix DNA-binding protein is essential for self-fertility during sexual development of the homothallic fungus *Fusarium graminearum*. mSphere. (2024) 9:e00511:–24. doi: 10.1128/msphere.00511-24, PMID: 39189781 PMC11423578

[52] Huang P Yu X Liu H Ding M Wang Z Xu J-R . Regulation of TRI5 expression and deoxynivalenol biosynthesis by a long non-coding RNA in *Fusarium graminearum*. Nat Commun. (2024) 15:1216. doi: 10.1038/s41467-024-45502-w, PMID: 38332031 PMC10853542

[53] Meek IB Peplow AW Ake C Jr Phillips T Beremand MN. Tri1 encodes the cytochrome P450 monooxygenase for C-8 hydroxylation during trichothecene biosynthesis in *Fusarium sporotrichioides* and resides upstream of another new tri gene. Appl Environ Microbiol. (2003) 69:1607–13. doi: 10.1128/AEM.69.3.1607-1613.2003, PMID: 12620849 PMC150100

[54] Koizumi Y Nakajima Y Tanaka Y Matsui K Sakabe M Maeda K . A role in 15-deacetylcalonectrin acetylation in the non-enzymatic cyclization of an earlier bicyclic intermediate in *Fusarium* trichothecene biosynthesis. Int J Mol Sci. (2024) 25:4288. doi: 10.3390/ijms25084288, PMID: 38673874 PMC11050026

[55] Tag AG Garifullina GF Peplow AW Ake C Jr Phillips T Hohn TM . A novel regulatory gene, Tri10, controls trichothecene toxin production and gene expression. Appl Environ Microbiol. (2001) 67:5294–302. doi: 10.1128/AEM.67.11.5294-5302.2001, PMID: 11679358 PMC93303

[56] Li C Liu C. Enantioselective effect of chiral fungicide prothioconazole on *Fusarium graminearum*: fungicidal activity and DON biosynthesis. Environ Pollut. (2022) 307:119553. doi: 10.1016/j.envpol.2022.119553, PMID: 35640724

[57] Wang Y Li B Shang H Ma R Zhu Y Yang X . Effective inhibition of fungal growth, deoxynivalenol biosynthesis and pathogenicity in cereal pathogen *Fusarium* spp. by cold atmospheric plasma. Chem Eng J. (2022) 437:135307

[58] Dhakal U. (2023). Population genomics analysis of US *Fusarium graminearum* isolates and identification of the genetic basis of fitness traits. Kansas State University.

[59] Ismail Y. Molecular interactions of arbuscular mycorrhizal fungi with mycotoxin-producing fungi and their role in plant defense responses. Canada: Universite de Montreal (2011).

[60] Cardoza RE McCormick SP Lindo L Kim H-S Olivera ER Nelson DR . A cytochrome P450 monooxygenase gene required for biosynthesis of the trichothecene toxin harzianum a in Trichoderma. Appl Microbiol Biotechnol. (2019) 103:8087–103. doi: 10.1007/s00253-019-10047-2, PMID: 31384992

[61] Shin JY Bui DC Lee Y Nam H Jung S Fang M . Functional characterization of cytochrome P450 monooxygenases in the cereal head blight fungus *Fusarium graminearum*. Environ Microbiol. (2017) 19:2053–67. doi: 10.1111/1462-2920.13730, PMID: 28296081

[62] Gao J Liu D Nguyen C McCormick SP Proctor RH Luo S . Biosynthesis of the central tricyclic skeleton of trichothecene mycotoxins. J Am Chem Soc. (2025) 147:10331–8. doi: 10.1021/jacs.4c16973, PMID: 40070048

[63] Khatibi PA Newmister SA Rayment I McCormick SP Alexander NJ Schmale DG III. Bioprospecting for trichothecene 3-O-acetyltransferases in the fungal genus *Fusarium* yields functional enzymes with different abilities to modify the mycotoxin deoxynivalenol. Appl Environ Microbiol. (2011) 77:1162–70. doi: 10.1128/AEM.01738-10, PMID: 21169453 PMC3067217

[64] Okubara P Blechl A McCormick S Alexander N Dill-Macky R Hohn T. Engineering deoxynivalenol metabolism in wheat through the expression of a fungal trichothecene acetyltransferase gene. Theor Appl Genet. (2002) 106:74–83. doi: 10.1007/s00122-002-1066-2, PMID: 12582873

[65] Wang Y Li J Wang X Wu W Nepovimova E Wu Q . Deoxynivalenol and its modified forms: key enzymes, inter-individual and interspecies differences in metabolism. Drug Metab Rev. (2022) 54:331–42. doi: 10.1080/03602532.2022.2088786, PMID: 35695207

[66] Alexander NJ McCormick SP Waalwijk C van der Lee T Proctor RH. The genetic basis for 3-ADON and 15-ADON trichothecene chemotypes in *Fusarium*. Fungal Genet Biol. (2011) 48:485–95. doi: 10.1016/j.fgb.2011.01.003, PMID: 21216300

[67] Chen Y Kistler HC Ma Z. *Fusarium graminearum* trichothecene mycotoxins: biosynthesis, regulation, and management. Annu Rev Phytopathol. (2019) 57:15–39. doi: 10.1146/annurev-phyto-082718-100318, PMID: 30893009

[68] Xu C Wang J Zhang Y Luo Y Zhao Y Chen Y . The transcription factor FgStuA regulates virulence and mycotoxin biosynthesis via recruiting the SAGA complex in *Fusarium graminearum*. New Phytol. (2023) 240:2455–67. doi: 10.1111/nph.19297, PMID: 37799006

[69] Jiang C Zhang C Wu C Sun P Hou R Liu H . TRI6 and TRI10 play different roles in the regulation of deoxynivalenol (DON) production by cAMP signalling in *Fusarium graminearum*. Environ Microbiol. (2016) 18:3689–701. doi: 10.1111/1462-2920.13279, PMID: 26940955

[70] Kimura M Tokai T Takahashi-Ando N Ohsato S Fujimura M. Molecular and genetic studies of *Fusarium trichothecene* biosynthesis: pathways, genes, and evolution. Biosci Biotechnol Biochem. (2007) 71:2105–23. doi: 10.1271/bbb.70183, PMID: 17827683

[71] Deng Y You L Wang X Wu W Kuca K Wu Q . Deoxynivalenol: emerging toxic mechanisms and control strategies, current and future perspectives. J Agric Food Chem. (2023) 71:10901–15. doi: 10.1021/acs.jafc.3c02020, PMID: 37437258

[72] Wang Z Wu Q Kuča K Dohnal V Tian Z. Deoxynivalenol: signaling pathways and human exposure risk assessment—an update. Arch Toxicol. (2014) 88:1915–28. doi: 10.1007/s00204-014-1354-z, PMID: 25199684

[73] Yun Y Liu Z Yin Y Jiang J Chen Y Xu JR . Functional analysis of the *Fusarium graminearum* phosphatome. New Phytol. (2015) 207:119–34. doi: 10.1111/nph.13374, PMID: 25758923

[74] Duan K Qin S Cui F Zhao L Huang Y Xu J-R . MeJA inhibits fungal growth and DON toxin production by interfering with the cAMP-PKA signaling pathway in the wheat scab fungus *Fusarium graminearum*. MBio. (2025) 16:e03151:–24. doi: 10.1128/mbio.03151-24, PMID: 39902906 PMC11898702

[75] Hou S Ma J Cheng Y Wang H Sun J Yan Y. The toxicity mechanisms of DON to humans and animals and potential biological treatment strategies. Crit Rev Food Sci Nutr. (2023) 63:790–812. doi: 10.1080/10408398.2021.1954598, PMID: 34520302

[76] Hu S Zhou X Gu X Cao S Wang C Xu J-R. The cAMP-PKA pathway regulates growth, sexual and asexual differentiation, and pathogenesis in *Fusarium graminearum*. Mol Plant-Microbe Interact. (2014) 27:557–66. doi: 10.1094/MPMI-10-13-0306-R, PMID: 24450772

[77] Bian C Duan Y Xiu Q Wang J Tao X Zhou M. Mechanism of validamycin a inhibiting DON biosynthesis and synergizing with DMI fungicides against *Fusarium graminearum*. Mol Plant Pathol. (2021) 22:769–85. doi: 10.1111/mpp.13060, PMID: 33934484 PMC8232029

[78] Yu F Gu Q Yun Y Yin Y Xu JR Shim WB . The TOR signaling pathway regulates vegetative development and virulence in *Fusarium graminearum*. New Phytol. (2014) 203:219–32. doi: 10.1111/nph.12776, PMID: 24684168

[79] Gao X Mu P Wen J Sun Y Chen Q Deng Y. Detoxification of trichothecene mycotoxins by a novel bacterium, *Eggerthella* sp. DII-9. Food Chem Toxicol. (2018) 112:310–9. doi: 10.1016/j.fct.2017.12.066, PMID: 29294345

[80] Ramirez ML Chulze S Magan N. Temperature and water activity effects on growth and temporal deoxynivalenol production by two Argentinean strains of *Fusarium graminearum* on irradiated wheat grain. Int J Food Microbiol. (2006) 106:291–6. doi: 10.1016/j.ijfoodmicro.2005.09.004, PMID: 16236377

[81] Moraes W. B. (2021). Sampling for Fusarium head blight (FHB) index estimation and quantifying the effects of environmental conditions on FHB development, mycotoxin contamination of grain, and their management in wheat. The Ohio State University.

[82] Ding Y Gardiner DM Xiao D Kazan K. Regulators of nitric oxide signaling triggered by host perception in a plant pathogen. Proc Natl Acad Sci. (2020) 117:11147–57. doi: 10.1073/pnas.1918977117, PMID: 32376629 PMC7245131

[83] Merhej J Boutigny A-L Pinson-Gadais L Richard-Forget F Barreau C. Acidic pH as a determinant of TRI gene expression and trichothecene B biosynthesis in *Fusarium graminearum*. Food Addit Contam. (2010) 27:710–7. doi: 10.1080/19440040903514531, PMID: 20169482

[84] Nakajima Y Akasaka M Shiobara T Kitou Y Maeda K Kanamaru K . Impact of nitrogen metabolism-associated culture pH changes on regulation of *Fusarium trichothecene* biosynthesis: revision of roles of *Polyamine agmatine* and transcription factor AreA. Curr Genet. (2020) 66:1179–90. doi: 10.1007/s00294-020-01102-x, PMID: 32812074

[85] Merhej J Richard-Forget F Barreau C. The pH regulatory factor Pac1 regulates tri gene expression and trichothecene production in *Fusarium graminearum*. Fungal Genet Biol. (2011) 48:275–84. doi: 10.1016/j.fgb.2010.11.008, PMID: 21126599

[86] Jiao F Kawakami A Nakajima T. Effects of different carbon sources on trichothecene production and tri gene expression by *Fusarium graminearum* in liquid culture. FEMS Microbiol Lett. (2008) 285:212–9. doi: 10.1111/j.1574-6968.2008.01235.x, PMID: 18564338

[87] Amarasinghe CC Fernando WD. Comparative analysis of deoxynivalenol biosynthesis related gene expression among different chemotypes of *Fusarium graminearum* in spring wheat. Front Microbiol. (2016) 7:1229. doi: 10.3389/fmicb.2016.01229, PMID: 27550207 PMC4976091

[88] Wang C Li P Cong W Ma N Zhou M Hou Y. The transcription factor FgCreA modulates ergosterol biosynthesis and sensitivity to DMI fungicides by regulating transcription of FgCyp51A and FgErg6A in *Fusarium graminearum*. Int J Biol Macromol. (2025) 284:137903. doi: 10.1016/j.ijbiomac.2024.137903, PMID: 39581416

[89] Gardiner DM Kazan K Praud S Torney FJ Rusu A Manners JM. Early activation of wheat polyamine biosynthesis during Fusarium head blight implicates putrescine as an inducer of trichothecene mycotoxin production. BMC Plant Biol. (2010) 10:289. doi: 10.1186/1471-2229-10-289, PMID: 21192794 PMC3022911

[90] Hou R Jiang C Zheng Q Wang C Xu JR. The AreA transcription factor mediates the regulation of deoxynivalenol (DON) synthesis by ammonium and cyclic adenosine monophosphate (cAMP) signalling in *Fusarium graminearum*. Mol Plant Pathol. (2015) 16:987–99. doi: 10.1111/mpp.12254, PMID: 25781642 PMC6638501

[91] Ma T Zhang L Wang M Li Y Jian Y Wu L . Plant defense compound triggers mycotoxin synthesis by regulating H2B ub1 and H3K4 me2/3 deposition. New Phytol. (2021) 232:2106–23. doi: 10.1111/nph.17718, PMID: 34480757 PMC9293436

[92] Nasmith CG Walkowiak S Wang L Leung WW Gong Y Johnston A . Tri6 is a global transcription regulator in the phytopathogen *Fusarium graminearum*. PLoS Pathog. (2011) 7:e1002266. doi: 10.1371/journal.ppat.1002266, PMID: 21980289 PMC3182926

[93] Ponts N Pinson-Gadais L Barreau C Richard-Forget F Ouellet T. Exogenous H2O2 and catalase treatments interfere with tri genes expression in liquid cultures of *Fusarium graminearum*. FEBS Lett. (2007) 581:443–7. doi: 10.1016/j.febslet.2007.01.003, PMID: 17250833

[94] Jiang C Zhang S Zhang Q Tao Y Wang C Xu JR. FgSKN 7 and FgATF 1 have overlapping functions in ascosporogenesis, pathogenesis and stress responses in *Fusarium graminearum*. Environ Microbiol. (2015) 17:1245–60. doi: 10.1111/1462-2920.12561, PMID: 25040476

[95] Montibus M Ducos C Bonnin-Verdal M-N Bormann J Ponts N Richard-Forget F . The bZIP transcription factor Fgap1 mediates oxidative stress response and trichothecene biosynthesis but not virulence in *Fusarium graminearum*. PLoS One. (2013) 8:e83377. doi: 10.1371/journal.pone.0083377, PMID: 24349499 PMC3861502

[96] Oufensou S Balmas V Azara E Fabbri D Dettori MA Schüller C . Naturally occurring phenols modulate vegetative growth and deoxynivalenol biosynthesis in *Fusarium graminearum*. ACS Omega. (2020) 5:29407–15. doi: 10.1021/acsomega.0c04260, PMID: 33225172 PMC7676359

[97] Boutigny A-L Barreau C Atanasova-Penichon V Verdal-Bonnin M-N Pinson-Gadais L Richard-Forget F. Ferulic acid, an efficient inhibitor of type B trichothecene biosynthesis and tri gene expression in Fusarium liquid cultures. Mycol Res. (2009) 113:746–53. doi: 10.1016/j.mycres.2009.02.010, PMID: 19249362

[98] Deng L-Z Sutar PP Mujumdar AS Tao Y Pan Z Liu Y-H . Thermal decontamination technologies for microorganisms and mycotoxins in low-moisture foods. Annu Rev Food Sci Technol. (2021) 12:287–305. doi: 10.1146/annurev-food-062220-112934, PMID: 33317321

[99] Pronyk C Cenkowski S Abramson D. Superheated steam reduction of deoxynivalenol in naturally contaminated wheat kernels. Food Control. (2006) 17:789–96. doi: 10.1016/j.foodcont.2005.05.004

[100] Vidal A Sanchis V Ramos AJ Marín S. Thermal stability and kinetics of degradation of deoxynivalenol, deoxynivalenol conjugates and ochratoxin a during baking of wheat bakery products. Food Chem. (2015) 178:276–86. doi: 10.1016/j.foodchem.2015.01.098, PMID: 25704712

[101] Khaneghah AM Moosavi MH Oliveira CA Vanin F Sant'Ana AS. Electron beam irradiation to reduce the mycotoxin and microbial contaminations of cereal-based products: an overview. Food Chem Toxicol. (2020) 143:111557. doi: 10.1016/j.fct.2020.111557, PMID: 32640360

[102] Li M He S Yang S Peng N Shen H Liu X . Deoxynivalenol removal and quality evaluation of foxtail millet under electron beam irradiation. Food Control. (2025) 174:111245. doi: 10.1016/j.foodcont.2025.111245

[103] Feizollahi E Arshad M Yadav B Ullah A Roopesh M. Degradation of deoxynivalenol by atmospheric-pressure cold plasma and sequential treatments with heat and UV light. Food Eng Rev. (2021) 13:696–705. doi: 10.1007/s12393-020-09241-0

[104] Shanakhat H Sorrentino A Raiola A Reverberi M Salustri M Masi P . Technological properties of durum wheat semolina treated by heating and UV irradiation for reduction of mycotoxin content. J Food Process Eng. (2019) 42:e13006. doi: 10.1111/jfpe.13006

[105] Jiang M Ji J Zhang Y Sun S. Removal of aflatoxins in peanut oils by activated carbon functionalized with sodium dodecyl sulfonate. Food Control. (2023) 153:109935. doi: 10.1016/j.foodcont.2023.109935

[106] Liu M Zhao L Gong G Zhang L Shi L Dai J . Invited review: remediation strategies for mycotoxin control in feed. J Anim Sci Biotechnol. (2022) 13:19. doi: 10.1186/s40104-021-00661-4, PMID: 35090579 PMC8796454

[107] Tu Y Liu S Cai P Shan T. Global distribution, toxicity to humans and animals, biodegradation, and nutritional mitigation of deoxynivalenol: a review. Compr Rev Food Sci Food Saf. (2023) 22:3951–83. doi: 10.1111/1541-4337.13203, PMID: 37421323

[108] Young JC Zhu H Zhou T. Degradation of trichothecene mycotoxins by aqueous ozone. Food Chem Toxicol. (2006) 44:417–24. doi: 10.1016/j.fct.2005.08.015, PMID: 16185803

[109] Sun X Ji J Gao Y Zhang Y Zhao G Sun C. Fate of deoxynivalenol and degradation products degraded by aqueous ozone in contaminated wheat. Food Res Int. (2020) 137:109357. doi: 10.1016/j.foodres.2020.109357, PMID: 33233060

[110] Yang Y Xu Y Wu S Qiu T Blaženović I Sun J . Evaluation of the toxicity and chemical alterations of deoxynivalenol degradation products under ozone treatment. Food Control. (2021) 124:107937

[111] Obadi M Zhu KX Peng W Sulieman AA Mahdi AA Mohammed K . Shelf life characteristics of bread produced from ozonated wheat flour. J Texture Stud. (2018) 49:492–502. doi: 10.1111/jtxs.12309, PMID: 29131335

[112] Bamyar E Peighambardoust SH Ghaziani MA. Bridging agriculture and food: aqueous ozone treatment optimizes wheat flour and dough rheology to enhance bread quality in Iranian cultivars. J Agric Food Res. (2025) 2025:102036

[113] Wang Y Hu J Dai Y Wang Y Shi J Wang G . Design and characterization of an artificial two-strain bacterial consortium for the efficient biodegradation of deoxynivalenol. Biol Control. (2023) 179:105172. doi: 10.1016/j.biocontrol.2023.105172

[114] Liu J Li P Li X Xie Y Mwabulili F Sun S . Expression, characterization, and application of an aldo-keto reductase mined from *Bacillus velezensis* Vel-HNGD-F2 for deoxynivalenol biodegradation. Food Chem Toxicol. (2025) 196:115159. doi: 10.1016/j.fct.2024.115159, PMID: 39613245

[115] Chen X Li Z Zhang X Zheng H Lv H Zhang W . Insights into the zearalenone degradation performance and pathway by *Gordonia hydrophobica* HAU421 and characterization of a novel lactonohydrolase involved. Int J Biol Macromol. (2025) 296:139631. doi: 10.1016/j.ijbiomac.2025.139631, PMID: 39793816

