# Correction: Recent advances in the biosynthetic mechanisms, regulation, and detoxification strategies of deoxynivalenol in *Fusarium graminearum*

**DOI:** 10.3389/fnut.2026.1774269

**Published:** 2026-01-23

**Authors:** Mingfang He, Yaping Lei, Haiyuan Yan, Chao Sun, Xiaolong Gu

**Affiliations:** 1 Modern College of Agriculture and Forestry Engineering, Ganzhou Polytechnic, Ganzhou, China; 2 College of Food Science and Engineering, Jiangxi Agricultural University, Nanchang, China; 3 College of Veterinary Medicine, Yunnan Agricultural University, Kunming, China

**Keywords:** Fusarium head blight, deoxynivalenol (DON), TRI gene cluster, biosynthesis, regulation, detoxification

Affiliation 1, “Modern College of Agriculture and Forestry Engineering, Ganzhou Polytechnic, Ganzhou, China”, was erroneously given as “Modern College of Agriculture, Forestry Engineering Ganzhou Polytechnic, Ganzhou, China”

The original version of the article has been updated.

